# Localized early mesenteric Castleman's disease presenting as recurrent intestinal obstruction: a case report

**DOI:** 10.1186/1746-1596-4-42

**Published:** 2009-12-04

**Authors:** Dina El Demellawy, Chaturika Herath, Francoise Truong, Ahmed Nasr, Salem Alowami

**Affiliations:** 1 Department of Pathology, Northern Ontario School of Medicine, Thunder Bay, Ontario, Canada; 2 Department of Laboratory Medicine, William Osler Health Center-Brampton Civic Hospital, Brampton, Ontario, Canada; 3 Department of Surgery, Sick Kids Hospital, University of Toronto, Toronto, Ontario, Canada; 4 Department of Pathology and Molecular Medicine, McMaster University, Hamilton Health Sciences Center, Hamilton, Ontario, Canada

## Abstract

Primary neoplasms of the mesentery are very rare. They are usually of mesenchymal origin and include desmoid tumor, lipoma, liposarcoma, and fibrosarcoma. Metastatic carcinomas and lymphoma are more common. We report a rare case of localized mesenteric Castleman's disease, presenting as intestinal obstruction. Clinical and radiological findings were suspicious for lymphoma. Localized mesenteric Castleman's disease, though rare, has to be considered in the differential diagnosis of mesenteric tumors, particularly in the young and in the absence of history for other tumor, an abnormal blood picture, or splenomegaly.

## Background

Primary neoplasms of the mesentery are very rare. They are usually of mesenchymal origin and include desmoid tumor, lipoma, liposarcoma, and fibrosarcoma. Metastatic carcinomas and lymphoma are more common. We report a rare case of localized mesenteric Castleman's disease, presenting as intestinal obstruction. Clinical and radiological findings were suspicious for lymphoma. Localized mesenteric Castleman's disease, though rare, has to be considered in the differential diagnosis of mesenteric tumors, particularly in the young and in the absence of history for other tumor, an abnormal blood picture, or splenomegaly.

## Case Presentation

### Clinical findings

A thirty-three year old female presented with recurrent symptoms of small intestinal obstruction of chronic onset and progressive course. The patient's medical history was non contributory. Her blood picture was unremarkable. CT scan showed intussusception associated with multiple mesenteric lymphadenopathies, measuring 1.8 cm in maximum dimension. Mediastinal lymphadenopathy and organomegaly were absent. The clinical impression was suspicious for lymphoma.

Laporotomy was performed which showed increased caliber and thickening of the small intestinal loops, particularly at the ileum over an area of about 90-100 cm. In addition, several enlarged mesenteric lymph nodes were present. An excisional biopsy from the nodes and a full thickness wedge excision from the abnormal area in the ileum were performed.

### Pathological findings

A piece of adipose tissue containing two lymph nodes measuring 2.1 × 1.5 × 1 cm and 1 × 1 × 1 cm and a small intestinal wedge measuring 4.5 cm long are received fresh. The lymph nodes are submitted according to the lymphoma protocol in our institute. Two touch preparations are prepared from the cut surface. The larger node is trisected and the smaller is bisected, with one section of each is submitted for flow cytometry assay. A tiny piece from the larger node is sent to microbiology. The rest of the tissue is submitted for formalin fixation. The intestinal wedge is opened and showed an intact, unremarkable wall. Representative sections are taken.

**Figure 1:**
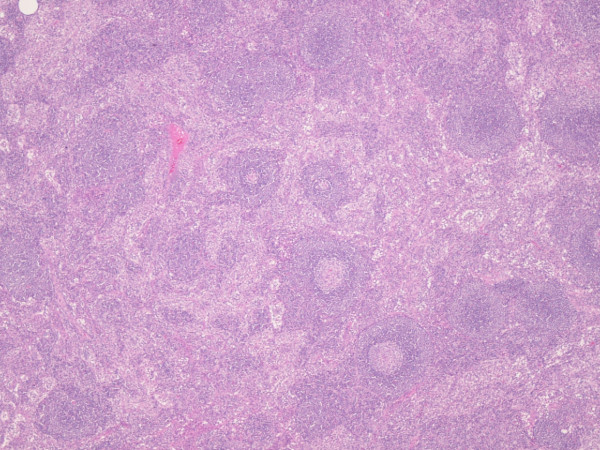
**Low power view showing lymph node with preserved architecture and reactive lymphoid follicles (HE ×100)**.

**Figure 2:**
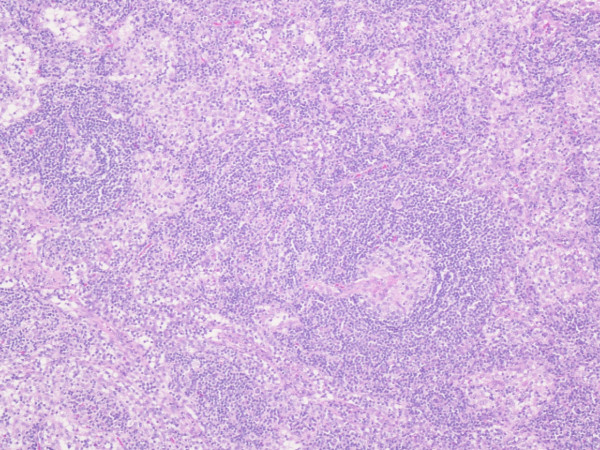
**High power view showing the typical appearance of the hyaline-vascular type of Castleman's disease, with small germinal centers of the lymphoid follicles traversed by a hyalinised blood vessel (HE ×400)**.

Microscopic examination shows the lymph nodes to have preserved architecture, but the lymphoid follicles are atrophic and possess a thickened mantle with prominent onion skin arrangement. Few follicles are traversed by a thick hyalinised blood vessel imparting a lollipop like appearance (Fig. 1 and Fig. 2). The interfollicular zone and paracortex are unremarkable. Plasmacytosis is absent. Flow cytometry assay is normal. Similarly the ileal wedge is within normal limits.

A diagnosis of early mesenteric Castleman's disease is made. The patient received no further treatment, and during the one year follow-up she is well.

## Conclusion

Castleman's disease (giant lymph node hyperplasia or angiofollicular lymphoid hyperplasia) is a rare lymphoproliferative disease of unknown origin, first described by Castleman's in 1954 [1]. Castleman disease is a benign condition of unknown etiology characterized by proliferation of mature lymphocytes and/or plasma cells.

Castleman's disease can be divided into two further forms: the most common localized-solitary form and the less usual is the multicentric form. Approximately 80% of the cases of the solitary form belong to the hyaline-vascular type and the remaining 20% to the plasma cell type.

The usual location of the solitary mass is the mediastinum (70%), whereas the mesentery is very rarely involved. The hyaline vascular type predominates in the thorax and is rare in the mesentery [2]. The enlarged lymph nodes are highly vascular [3]. The widespread form of the disease is characterized by disseminated lymphadenopathy, is almost always associated with systemic symptoms such as fever, hematologic disorders including anemia and hyperglobulinemia, endocrinopathies or peripheral neuropathies, and is dominated by the plasma cell type [3-5]. Association with other gastrointestinal diseases has been rarely reported [6].

Most Castleman's disease lesions appear as nonspecific, well-defined hypoechoic masses on sonography. Sonography remains useful for the evaluation of cervical and axillary Castleman's diseases, in which the depiction of prominent peripheral vessels and penetrating feeding vessels on Doppler sonograms can suggest the diagnosis of this uncommon disease [7,8]. On CT scan, Castleman's disease has been described as a well circumscribed homogeneous mass lesion with moderate to intense enhancement. The hyaline vascular type has a tendency to enhance more than the plasma cell type, due to the greater vascularity of the former [7-10]. Calcification can occur in up to 50% of the cases, particularly those located in the pelvis [11]. Unfortunately, these radiological features are not specific for Castleman's disease, as they can be observed in every lymphomatous tumor, benign or malignant, or other mesenteric masses. Hence the gold standard for diagnosis is pathological examination.

Castleman's disease has to be included in the differential diagnosis of every mass located in the mesentery, especially when the lesion shows the imaging features mentioned above, and if the patient is young, and if relevant medical history, an abnormal blood picture, and organomegaly are absent.

## Competing interests

The authors declare that they have no competing interests.

## Authors' contributions

DE: wrote the manuscript.

CH: made the corrections as for the reviewer's comments.

FT: the case was originally diagnosed by FT and reviewed by DE through intra-departmental review.

AN: took the pictures and wrote legends.

SA: revised and provided the publication fees.
